# Challenges in the Design of a T Cell Vaccine in the Context of HIV-1 Diversity

**DOI:** 10.3390/v6103968

**Published:** 2014-10-23

**Authors:** Marcel Tongo, Wendy A. Burgers

**Affiliations:** 1 Institute of Infectious Disease and Molecular Medicine, Division of Medical Virology, University of Cape Town, Anzio Road, Observatory 7925, Cape Town, South Africa; E-Mail: wendy.burgers@uct.ac.za; 2 Institute of Medical Research and Study of Medicinal Plants (IMPM), Route de l’École Normale Supérieure, Yaoundé, Cameroon; 3 Current address: Cellular Immunology Group, International Centre for Genetic Engineering and Biotechnology (ICGEB), University of Cape Town, Anzio Road, Observatory 7925, Cape Town, South Africa

**Keywords:** HIV-1, diversity, broadly cross-reactive T cell responses, vaccine, immunogen design

## Abstract

The extraordinary variability of HIV-1 poses a major obstacle to vaccine development. The effectiveness of a vaccine is likely to vary dramatically in different populations infected with different HIV-1 subtypes, unless innovative vaccine immunogens are developed to protect against the range of HIV-1 diversity. Immunogen design for stimulating neutralizing antibody responses focuses on “breadth” – the targeting of a handful of highly conserved neutralizing determinants on the HIV-1 Envelope protein that can recognize the majority of viruses across all HIV-1 subtypes. An effective vaccine will likely require the generation of both broadly cross-neutralizing antibodies and non-neutralizing antibodies, as well as broadly cross-reactive T cells. Several approaches have been taken to design such broadly-reactive and cross-protective T cell immunogens. Artificial sequences have been designed that reduce the genetic distance between a vaccine strain and contemporary circulating viruses; “mosaic” immunogens extend this concept to contain multiple potential T cell epitope (PTE) variants; and further efforts attempt to focus T cell immunity on highly conserved regions of the HIV-1 genome. Thus far, a number of pre-clinical and early clinical studies have been performed assessing these new immunogens. In this review, the potential use of these new immunogens is explored.

## 1. Introduction

The AIDS pandemic is more than 30 years old. There were 2.3 million new HIV-1 infections in 2012, 33% fewer than in 2001 [1]. Much of this success has been recorded in the last four years, and can be attributed to the rapid improvement in access to effective antiretroviral therapy (ART). In addition to the success of ART, there have been a number of other achievements in the goal to reduce the burden of HIV/AIDS. Several prevention methods have proved efficacious in recent years, such as voluntary medical male circumcision (MMC) [2,3], pre-exposure prophylaxis (PrEP), which involves HIV-uninfected persons taking some of the same anti-HIV-1 drugs as those used to treat infected people [4,5], and ART as prevention, where treatment of the infected partner in discordant couples leads to a high level of protection for the uninfected partner [6]. Although full implementation of these preventive measures has the potential to drastically reduce the spread of HIV-1, they invariably face the huge challenges of cost and behavior change, including life-long, daily adherence. Therefore, a highly efficacious vaccine that would prevent HIV-1 infection and/or progression to AIDS remains an urgent need. The development of such a globally-effective vaccine faces many challenges, including the considerable genetic diversity that HIV-1 exhibits, the incomplete knowledge of correlates of a protective immune response against the virus, and how to generate such a response with a vaccine. Understanding these challenges is of great importance in the design of an effective HIV-1 vaccine. This review provides an overview of the key concepts in HIV-1 diversity, immunity and immunogen design pertinent to the development of a T-cell inducing HIV-1 vaccine.

## 2. HIV-1 Diversity

One of the most important characteristics of HIV-1 is its extreme genetic diversity. There are two viral mechanisms that generate HIV-1 diversity: (1) mutations are introduced into viral genomes during replication by an error-prone reverse transcriptase enzyme; and (2) mutations are introduced by recombination between viral genomes. There are presently four groups of HIV-1 likely resulting from independent cross-species transmission to humans [7,8,9,10]. HIV-1 group M (Main or Major) is responsible for the global epidemic [11,12]. The O (Outlier) group, which for unknown reasons has largely remained endemic to Cameroon, is responsible for 1%–5% of HIV-1 infections [13,14,15]. Even more rare, group N (Non-M and non-O) is only found in Cameroon or in Cameroonian individuals [16,17], and group P has also only been isolated in two patients from Cameroon [18,19]. HIV-1 group M can be subdivided into subtypes based on their phylogenetic relatedness. Currently there are nine described subtypes named A, B, C, D, F, G, H, J and K [6]. Two of these, subtypes A and F, have been further subdivided into sub-subtypes (referred to as A1, A2 and F1, F2 respectively) [11]. Other significant clusters are formed by circulating recombinant forms (CRFs). These have arisen as a result of recombination events between divergent HIV-1 strains within individual hosts [11]. Their number is constantly changing, and there are currently more than 50 CRFs. When CRFs are found in isolated individuals, they are termed unique recombinant forms (URFs). The currently classified HIV-1 subtypes and CRFs are representative of the viruses primarily responsible for the AIDS epidemic. However, even this enormous variability does not give a complete view of HIV-1 diversity. There are presently many fully sequenced HIV-1 group M viruses that are too divergent to be placed within any existing subtype or CRF grouping, and these have remained unclassified [20]. Recently, we have characterized HIV-1*gag* and *nef* genetic diversity in 59 plasma samples from HIV-1-infected blood donors from Cameroon; we found that five *gag* sequences (10%) and three *nef* sequences (5%) were neither obviously recombinant nor easily classifiable into any of the known HIV-1 group M subtypes [21]. In addition, certain inter-subtype recombinant viruses contain sequences that are of indeterminate origin, providing further evidence that HIV-1 diversity is not fully represented under the current classification system [20]. This implies that there is potentially a far more diverse pool of HIV-1 sequences circulating amongst humans than the classified subtypes and CRFs might suggest. It is likely that cross-species transmission took place in equatorial West Africa, and specifically in southern Cameroon, habitat of western gorillas and chimpanzees [9,10]. After transmission to humans, HIV-1 group M began to diversify. The greatest genetic diversity of HIV-1 group M in terms of number of subtypes and genetic diversity within subtypes has been observed in the western region of the Democratic Republic of Congo (DRC), suggesting that this was the epicenter of the epidemic [22,23].

The overall variability of the virus is further complicated by a complex mixture of viral populations or quasispecies, closely related but not identical, which vary continuously under immune pressure. For example, Korber *et al.* [24] demonstrated that the variability of HIV-1 within one host is comparable to the global variation of influenza A. The mutability of HIV-1 readily allows it to escape the neutralizing antibody and T cell responses of the host during the course of infection [25,26]. This phenomenon has been well documented in SIV-vaccinated macaques, where CD8^+^ T cell escape variants have led to the vaccine failure [27,28].

## 3. T Cell Immunity to HIV-1

Early studies demonstrated that HIV-1-infected people mount vigorous CD8^+^ T cell responses to the virus [29,30], and these responses were considered as potential effectors for future HIV-1 vaccines [31,32]. Understanding the dynamics of cellular immune responses in natural HIV-1 infection in humans and SIV infection in animal models has been the topic of much study over the last 20 years [33,34,35]. These studies provided strong evidence that CD8^+^ T cells are important in controlling virus replication during HIV infection, which led to the testing of the “T cell concept” in clinical trials of HIV-1 vaccine candidates. Although these trials were spectacularly disappointing, the T cell concept has recently been revived, with alternative vaccination approaches appearing more promising, discussed below.

### 3.1. The Role of T Cells in the Control of HIV-1

Several arguments underscore the essential role of the CD8^+^ T cell response in controlling viral replication during HIV-1 infection. These include the parallel decrease of HIV-1 viral load with the peak of the CD8^+^ T cell response during the acute phase of infection [36], the rapid clearance of the transmitted virus strain [37], the loss of control of SIV infection in macaques after removal of their CD8^+^ T cells [38,39] and the association of particular HLA class I alleles with better control of the infection [40,41]. Thus, the search for the characteristics of HIV-1-specific CD8^+^ T cell responses that are associated with viral control, including the quantity, specificity and functional and phenotypic nature of the response, would certainly help with designing an effective HIV-1 vaccine.

The first studies on the quantity of HIV-1-specific T cell responses examined their magnitude and breadth, in an attempt to determine how these parameters were associated with clinical measures of disease. Addo *et al.* [42], using an interferon-gamma enzyme-linked immunospot (IFN-γ ELISPOT) assay, screened HIV-1-infected individuals for virus-specific T cell responses using peptides spanning all HIV-1 proteins. Despite broad and strong HIV-1-specific responses amongst these individuals, neither the breadth nor the magnitude of the total HIV-1-specific CD8^+^ T cell response was associated with plasma viral load [42]. These findings were replicated in many other studies, including cohorts from subtype C-infected individuals from the large southern African epidemic [43]. The majority of these studies were performed in a cross-sectional manner, with infected individuals at different stages of infection. Follow up studies using the same methodology on longitudinal samples, including those from early infection, also found no association with the rate of CD4^+^ T cell decline or the course of disease in the first year of infection [44,45]. Collectively, the conclusion drawn from these studies was that most HIV-1-infected individuals are able to mount robust CD8^+^ T cell IFN-γ responses and that the overall breadth and magnitude of these responses was not a correlate of viral control. These studies also suggested that other features, including the specificity (*i.e.*, which regions of HIV-1 were targeted) or the quality (e.g., effector functions besides IFN-γ) of the response may be important for viral control.

Several studies went on to demonstrate that the targeting of the HIV-1 Gag protein was associated with lower viral loads. Using the IFN-γ ELISPOT assay, it was found that the magnitude and breadth of T cell responses directed to Gag correlated inversely with viral load and directly with absolute CD4^+^ T cell counts, whilst no such associations were detected with other HIV-1 proteins [46]. Consistent with this finding, Masemola *et al.* [47] showed that the hierarchical targeting of the HIV-1 Gag, rather than the overall magnitude of T cell responses to HIV-1 proteins, was associated with viral control. In a more extensive study to define the relationship between the specificity of the CD8^+^ T cell response and viral control, Kiepiela *et al.* [48] provided evidence that a greater breadth of Gag-specific CD8^+^ responses was associated with lower viral load, while Env-specific responses were associated with higher viraemia. This association was independent of major histocompatibility complex class one (MHC-1) type and unrelated to epitope sequence conservation. These data demonstrate that Gag is an essential feature for any HIV-1 vaccine designed to elicit protective CD8^+^ T cell responses. Recently, these observations have been extended to other HIV proteins. Specific Nef epitopes have been linked with SIV control in macaques [49] and HIV in viral controllers, particularly when targeting critical functional regions of the protein [50,51,52,53]. These studies, therefore, suggest that other proteins besides Gag can also play a role in the control of viral replication, and that this should guide the design of vaccine immunogens.

The utility of the IFN-γ ELISPOT assay as a method for the quantification of the HIV-1-specific T cell responses was challenged, due to the lack of efficacy of a recently conducted clinical trial of an HIV-1 vaccine, the Step study, where ELISPOT responses of meager breadth and magnitude were induced, and no efficacy was observed [54]. This finding, along with advances in multiparameter flow cytometry and development of assays testing direct antiviral capacities of T cells, directed researchers to examine the immune response to HIV-1 in more depth, by assessing the quality rather than the quantity of virus-specific T cell responses. Efforts at elucidating qualitative features of T cell responses that control viral replication have been performed mainly by studying of individuals who control virus without ART. In a seminal study, Betts *et al.* [55] compared the capacity of CD8^+^ T cells from HIV-1 progressors and nonprogressors to produce five different functions, namely secretion of the cytokines IFN-γ, IL-2, TNF-α, MIP1β, and expression of CD107a as a surrogate for degranulation and cytotoxicity. Polyfunctionality of CD8^+^ T cells, or their capacity to produce three or more cytokines simultaneously, was associated with control of viral replication, and nonprogressors consistently maintained highly functional CD8^+^ T cells [55]. Beyond polyfunctionality, cytotoxicity appears to be vital to the control of HIV-1 replication. Hersperger *et al.* [56] measured perforin upregulation, cytokine production, and degranulation after stimulation with peptide pools to all HIV-1 proteins in individuals who controlled and those who did not control viral replication. They observed that CD8^+^ T cells from controllers produced significantly higher perforin and granzymes than progressors [56]. Dissection and characterization of different combinations of functions in these HIV-1-specific T cells generated suggested that highly functional HIV-1-specific T cells were distinguished by perforin and IL-2 upregulation [56]. Another qualitative feature of CD8^+^ T cells, their proliferative capacity, was inversely associated with viral load [57]. A recent study demonstrated that the proliferative capacity of HIV-1-specific CD8^+^ T cells correlated with delayed disease progression, while the magnitude of IFN-γ responses did not [58]. In an effort to combine quantitative and qualitative measures of immunity, Riou *et al.* [59] evaluated the impact of the quantity, quality and specificity of CD8^+^ T cell responses at approximately six months post-infection on the viral set point at 12 months in a cohort of HIV-1-infected individuals in South Africa. A high frequency of highly functional Gag- and Nef-specific CD8^+^ T cell responses was the best predictor of a low viral set point [59]. Functional avidity, the capacity of T cells to respond to low concentrations of peptide, and differentiation phenotype of HIV-1-specific T cells, have also been examined. HIV-1 Gag-specific T cell responses in controllers and non-controllers was analyzed to determine the functional avidity of these responses. Although responses to p24 Gag were of comparable breadth and magnitude, significantly higher avidity responses were observed in controllers compared to non-controllers [59]. The maturation phenotype of CD8^+^ T cells is also likely to influence viral control. Fully differentiated HIV-1-specific CD8^+^ effector cells were seen to be more frequently detectable in controlled than in progressive HIV-1 infection [60,61,62], whilst central memory CD8^+^ T cells were associated with lower viral set points in early infection in another study [63]. A further advancement in our understanding of T cell immunity on control of HIV-1 has been the development of *in vitro* assays to determine the capacity of CD8^+^ T cells to directly inhibit viral replication [64,65]. In ELISPOT and flow cytometric assays, peptides are added in excess and presented without the need for endogenous processing, and several studies have demonstrated whilst the CD8^+^ T cells detected in these assays recognize HIV (or SIV), they may not necessarily have any effect on HIV-1 replication [66,67]. Using a viral inhibition assay (VIA), it has been demonstrated that the quality of CD8^+^ T cells influences the rate of HIV-1 progression, as the antiviral activity of CD8^+^ T cells in this assay was strongly predictive of the rate of CD4^+^ T cell decline [68,69]. This has been demonstrated in acute HIV infection [64], HIV controllers [70] and vaccine trials [65,71].

### 3.2. Putting the T Cell Vaccine Concept to the Test

In view of the potential role of CD8^+^ T cell responses in the control of HIV-1 replication, vaccines that elicited these responses were developed. Several types of HIV-1 vaccine candidates capable of inducing T cell responses have been tested over the past decade, with the most prominent being the Step trial. The Step trial was a Phase IIb study that involved 3000 volunteers at risk of HIV-1 infection, mostly from the Americas [54]. The goal of the trial was to determine whether the vaccine could prevent HIV-1 infection, or reduce viral load in those who became infected. The vaccine regimen consisted of an Adenovirus serotype 5 (Ad5) vector expressing the *gag*, *pol* and *nef* genes of HIV-1 subtype B. The Phambili trial of the same vaccine also entered phase IIb evaluation in South Africa, to explore whether it would also be effective at preventing infection from HIV-1 subtype C. The Step study was stopped due to lack of efficacy and a higher incidence of infections in the vaccinees, followed by a premature halting of the Phambili trial. The two trials showed that the vaccine failed to prevent HIV-1 infection or lower viral load set point [54,72,73]. However, 77% of vaccinees mounted CD8^+^ T cell responses to the vaccine, detected by ELISPOT [74,75,76]. Nevertheless, the sieve analysis, which compared breakthrough viruses between vaccinees and placebo recipients during the trial, suggested that T cell responses exerted pressure on founder viruses [77,78]. In addition, Gag-specific CD8^+^ T cell responses generated after vaccination were associated with reduced plasma viremia [79].

The recent Phase IIb HVTN505 trial, using a DNA prime vaccination and recombinant Ad5 boost, expressing *gag*, *pol*, *nef* and *env* genes (the later from three subtypes), was performed in high-risk individuals in the United States. The vaccine regimen was well tolerated but was also halted because of lack of efficacy [80]. Notably, the vaccine did not reduce the viral load set point in vaccinees that became infected on the trial [80].

### 3.3. New Evidence of Protective T Cell Immunity to HIV

Although the RV144 trial demonstrated moderate success in preventing HIV infection [81], there was no impact on measures of disease progression (viral load or CD4 cell count) in vaccinated volunteers who became infected [82], as with the other vaccine efficacy trials. This implies that a vaccine able to control HIV once an individual has become infected will very likely be extremely difficult to achieve. Thus, efforts to better understand the role of T cell immunity in the control of HIV-1 remain intense. Alternative vectors for HIV vaccines, with the goal of eliciting HIV-1-specific cellular immune responses, are now being developed based on less prevalent human adenovirus types, or on simian adenoviruses for which no pre-existing immunity exists in human populations. A replication-competent viral vector that has attracted much attention recently is cytomegalovirus (CMV), as it can elicit potent and long lasting CD4^+^ and CD8^+^ T effector memory (Tem) responses [83], functional attributes associated with viral control, as discussed previously in Section 3.1. Furthermore, Hansen *et al.* [84] showed that by genetically modifying CMV vectors, it is possible to program them to achieve distinct patterns of CD8^+^ T cell recognition to diverse and highly promiscuous epitopes. Remarkable vaccine-mediated protection in rhesus macaques was obtained with a replicating simian CMV vector expressing the SIV *gag*, *rev*, *tat*, *nef* and *env* genes [85]. The vaccine efficiently induced effector memory CD4^+^ and CD8^+^ tissue-resident T cells, which did not protect the animals against infection following low-dose rectal challenge, but elicited early and profound control of virus replication, with half of the monkeys showing viral loads below detection level [85]. These findings were extended and clearance of infection was confirmed in additional vaccinated and challenged monkeys [86]. They showed that infected macaques, regardless of the route of challenge, lost signs of SIV infection over time. In another study, Barouch *et al.* [87] assessed the protective efficacy of Ad/poxvirus and Ad/Ad-vector-based vaccines expressing SIV *gag*, *pol* and *env*, against neutralization-resistant SIV strains. Specific correlates of protection from SIV acquisition were identified, including magnitude and breadth of SIV-specific T cell responses. These results pave the way for the further development of a candidate vaccine that can elicit T cell immune responses in humans.

## 4. HIV-1 Vaccines

### 4.1. HIV-1 Vaccine Development

The development of an HIV-1 vaccine is an enormous task. Immunization has been successful in the eradication or elimination of some viral diseases, such as smallpox, polio and measles. Many traditional vaccines have been developed using live attenuated forms of virus, inactivated (killed) virus or protein subunits [88]. These approaches are not suitable for developing a vaccine against HIV-1. Attenuated HIV-1 may mutate and regain its pathogenicity after inoculation [89]. Inactive HIV-1 on the other hand may still contain enough live viruses to pose a risk [90]. Finally, subunit vaccines to HIV-1 envelope monomers were shown not to be protective in two large Phase III efficacy trials in humans, though protein boosts were used in the only HIV vaccine trial that to date has demonstrated any efficacy for protecting against infection [91,92,93]. Thus, challenges to developing an HIV-1 vaccine are complex and immense, and include not only the high genetic variability of the virus, the difficulty in generating broadly neutralizing antibody responses, unknown correlates of protection, but also the design of immunogens, limitations of animal models, and the difficulty in performing large clinical trials.

### 4.2. T Cell Vaccine Immunogens

Selection of immunogens in vaccine development remains a crucial issue, as we do not know the true extent of the influence of HIV-1 diversity on immune responses. Various approaches have been taken to contend with diversity, including the development of vaccine candidates with single natural sequences or sequences from multiple subtypes, centralized and mosaic sequences, and conserved epitopes.

#### 4.2.1. Single or Multi-Subtype Vaccines

Many vaccines have been developed with sequences that match the circulating HIV-1 subtype(s) in a particular region, with the hope of maximizing the chances of developing T cell responses to that subtype, and sufficient cross-reactivity against heterologous subtypes (cross-clade responses). Cross-clade responses, or immune responses generated by infected individuals to heterologous HIV-1 subtypes, have been demonstrated [93,94,95,96,97]. In an extensive study on cross-clade immune responses, Coplan *et al.* [98] determined cellular immune responses to HIV-1 subtypes A, B and C peptides in IFN-γ ELISPOT assays. In a large cohort of HIV-1-infected individual from four continents, with subtype A, B and C epidemics, cross-reactivity of cellular immune responses was observed for all HIV-1 proteins tested [98]. Further analyses from the same cohort extended to more HIV-1-infected individuals demonstrated similar extensive cross-reactivity among the three HIV-1 subtypes tested for Gag and Nef [99]. Cross-reactivity, measured by the number of infected individuals who reacted to heterologous peptides, was 99.1% between subtype C-infected individuals and subtype B Gag proteins, and 97.8% between subtype A-infected individuals and subtype B Gag proteins. In another study involving 39 subtype C-infected individuals, the magnitude and breadth of IFN-γ Gag-specific T cell responses were assessed for reactivity to five sets of overlapping peptides, two sets matching subtype C strains from South Africa and China, and three peptide sets corresponding to consensus subtypes A, B, and D sequences. Out of a total of 84 peptides that were recognized, 17 were commonly shared by subtypes A, B, C and D [93]. When taken together, these studies demonstrate that HIV-1-specific T cells from individuals infected with a particular HIV-1 subtype can extensively cross-recognize HIV-1 proteins or peptides based on other HIV-1 subtypes. These types of studies, however, have a number of limitations for extrapolating “cross-reactivity” to vaccine trials. These include testing cross-reactivity in individuals that have been infected with HIV for many years, and immune responses generated in natural infection might differ in magnitude, breadth, depth and quality from those generated by a vaccine, and, as discussed earlier, the use of the ELISPOT assay with non-physiological amounts of peptide used to detect responses *in vitro*, and whether these cross-reactive responses reflect cross-protection.

The phase IIb Step and Phambili trials tested a vaccine based on genes from HIV-1 subtype B [54]. These trials were performed in the Americas and Australia, with primarily a subtype B epidemic, and in South Africa, with a subtype C epidemic [54,72]. Unfortunately, the vaccine was not effective, so no insights could be gained on cross-protection. A subtype-specific vaccine was also tested in the RV144 trial, which used in a prime-boost strategy, subtype B and CRF01_AE genes, the infecting subtypes in Thailand where the trial was conducted [100]. The multi-subtype approach was tested in the phase IIb HVTN505 trial, which included *env* gene sequences from subtypes A, B and C [80]. The trial showed no efficacy against HIV-1. Since RV144 has been the only advanced trial that has shown any efficacy in preventing HIV infections, plans are underway to perform a confirmatory study in Thailand, as well as test the same vaccination concept in Southern Africa, substituting subtype C sequences to match the epidemic in this region (the P5 partnership [101]).

#### 4.2.2. Centralized Sequences

Different strategies have been proposed to tackle the huge genetic variability of the HIV-1 in vaccine immunogen design. One of these approaches is the design of centralized HIV-1 sequences that are based on a single subtype or on all group M viruses. This includes the most recent common ancestor (MRCA) sequence, which represents the ancestor from which a given group of sequences have descended [102,103], the center-of-tree sequence (COT), which is a sequence whereby the average evolutionary distance to each tip of a phylogeny tree is minimized [104,105,106], and a consensus sequence, consisting of the most common amino acids found at a given position in a group of sequences [105,107]. In contrast to the single subtype vaccine approaches, these approaches attempt to increase the breadth of responses to different subtypes.

To date, there have been no advanced vaccine trials in humans that have tested and compared the efficacy of these centralized immunogens in different regions of the world [108]. However, these sequences have been tested as candidate vaccines in preclinical studies, with most of these data generated with Env protein sequences. Mice vaccinated with DNA followed by recombinant vaccinia virus expressing consensus M gp120 and gp140, elicited T cell responses that targeted epitopes from subtype B and C [109]. In addition, Weaver *et al.* [110] found a similar magnitude and breadth of immune responses when comparing group M consensus Env immunogens with a B/C recombinant virus isolate in mice. These studies and others [107,111] have clearly demonstrated at least similar HIV-1-specific T cell responses are elicited compared to immunogens based on a single or multiple subtypes in mice.

In humans, in the absence of clinical trials testing centralized sequences, numerous studies have characterized the immunological recognition of HIV-1 centralized peptides in HIV-1-infected individuals. In a study involving HIV-1 subtypes B and C chronically-infected individuals from the US and South Africa, respectively, Bansal *et al.* [112] measured T cell immune responses to consensus Gag from subtype A, B, C and group M peptides and to ancestral subtype B and group M peptides using the IFN-γ ELISPOT assay. They demonstrated a similar broad cross-reactivity among the different peptides tested in both B and C epidemics. Additionally, Malhotra *et al.* [113] also showed a comparable magnitude and breadth of immune responses among subtype-specific peptides and group M peptides, all based on centralized sequences. On the other hand, Frahm *et al.* [114] found that group M peptides were less frequently targeted compared to subtype B peptides in a subtype B epidemic, whereas there was a similarity in the frequency of responses between group M reagents and subtypes C peptides in the same study involving subtype C-infected individuals. Along the same lines, a similar level of responses was detected with group M-based peptides in subtype B and F infected subjects, whereas this set of peptides detected a lower level of responses than consensus C among subtype C infected subjects [115]. Overall, these studies suggest that centralized sequences, and particularly group M peptides, were able to detect broad T cell responses. At the same time, these studies also emphasize the need to test centralized reagents in other epidemics, as these results may not be directly applicable to areas with different subtypes present, or with a large degree of HIV-1 diversity, such as West Central Africa. Recently, we investigated the extent of the diversity on T cell immune responses using HIV-1 group M consensus Gag and Nef peptides in a multiclade epidemic, namely Cameroon, where virtually all group M subtypes are present [21]. Compared with a monoclade C epidemic in South Africa, we showed that few epitopes were commonly targeted in the two epidemics, especially in Gag, where less than one third of all reactive peptides were commonly recognized. In addition, there was a clear signal of preferential targeting in the different populations, with some peptides been recognized at a high frequency in one population and not in the other population, with HLA diversity also a likely contributor to this differential targeting [116]. The central nature of HIV-1 consensus M sequences resulted in their broad recognition, but failed to identify highly immunodominant peptides between homogeneous and diverse HIV-1 epidemics. One limitation of this approach is that centralized sequences are generated with available sequences and need permanent updating; many regions of the world have few sequences available in HIV sequence databases.

#### 4.2.3. Mosaic Sequences

HIV-1 mosaic antigens are bioinformatically-optimized immunogens that maximize the coverage of natural variation of the virus, and also take into account the diverse MHC class I haplotypes [117]. They were developed by combining sequences from different HIV-1 subtypes using artificial recombination methods designed to mimic the recombination process that occurs during natural HIV-1 evolution [118]. This can be performed for a single subtype or for all the group M variants. Vaccines based on this approach utilize computerized algorithms to generate optimized sequences similar to naturally circulating HIV-1 sequences [118]. They are intended to demonstrate a greater coverage of HIV-1 potential T cell epitopes (PTEs) for different HIV-1 proteins [119]. Preliminary data on this approach suggests that the mosaic approach provides enhanced coverage of 9-mer peptides compared to the COT approach [119]. In contrast to the centralized approaches, the mosaic approach attempts to increase not only the breadth of the response, but also the “depth”, *i.e.*, the targeting of multiple variants of the same epitope.

Several vaccine candidates using the mosaic approach have been tested in preclinical studies. In mice, DNA vaccines expressing mosaic Env antigens were compared to natural Env strains. Mosaic candidates elicited responses to multiple variants of different epitopes, and this led to an increase in the breadth of response; an average of eight peptide pools, compared to two pools for a set of natural Env sequences [120]. Similar observations were made in rhesus macaques. Barouch *et al.* [121] immunized macaques with an Ad26 vector expressing mosaic HIV-1 Gag, Pol and Env antigens, M consensus, combined subtypes B and C, or natural subtype C sequences. The total number of Gag-, Pol- and Env-specific cellular responses elicited by the mosaic antigens was four-fold higher than the number of responses induced by the consensus or natural sequence antigens. More importantly, the mosaic vaccine induced T cell responses that recognized more variants within an epitope (depth) than consensus or natural sequence antigens [121]. A second similar preclinical study compared responses in animals receiving either mosaic or consensus group M Gag and Nef immunogens delivered by a DNA prime–recombinant vaccinia virus boost regimen [122]. Here, a greater magnitude, breadth and depth of responses to Gag mosaic immunogens were observed compared to consensus, but no difference was seen for Nef [122].

No vaccine candidates expressing mosaic immunogens have been tested in humans to date. In the absence of clinical trials evaluating these new vaccine immunogens, it is important to understand the responses that T cell vaccines based on these mosaic sequences could elicit or detect. As with natural or centralized sequences, one of the ways to achieve this is to evaluate the recognition of peptides based on the mosaic immunogens (PTE peptide sets) in HIV-1-infected individuals. The ability of a subtype B PTE peptide set based on Nef to detect T cells from HIV-1 subtype B-infected individuals has been tested [123]. T cells responses were evaluated with subtype B consensus and PTE Nef peptides in 23 individuals using the IFN-γ ELISPOT assay. Although the specificity of responses was comparable when the two sets of peptides were used, both the breadth and the magnitude of responses detected were significantly higher when peripheral blood mononuclear cells (PBMC) were stimulated with PTE peptides compared to consensus peptides. In addition, T cells able to cross-detect multiple variants of Nef were also induced [123]. Taking together, it is likely that the higher magnitude and breadth of responses to PTE against consensus or natural strains peptides could be explained by the increased proportion of recognized peptides with targeted variants. We have performed a similar study in a diverse epidemic in Cameroon comparing reactivity of Gag and Nef PTE peptides and group M peptides, and found that whilst we detected a significantly greater magnitude of T cell responses with the PTE peptide set than with the consensus M set in Gag, the magnitude of responses was similar between the two sets of peptides in Nef; so too, the breadth of responses was similar between the two set of peptides both in Gag and Nef. Recognition of multiple variants was detected with the PTE peptide set, with up to five variants being recognized for particular peptides. This was more pronounced in Gag where out of the total peptides targeted, 60% had also at least one variant recognized [124]. This underscores the fact that there are fewer characterized epitopes from the diverse set of viruses circulating in Cameroon and in west central Africa present in HIV sequence databases, so if a mosaic approach for an HIV-1 vaccine is pursued, or the use of PTE peptides for vaccine testing is implemented, additional mosaic sequences may need to be included for greater coverage in highly diverse epidemics.

The extent of HIV-1 diversity is extreme, and it is not known whether mosaic HIV vaccines will induce targeting of viral epitopes relevant to HIV control. Planned trials in the pipeline using these immunogens [108] will ultimately determine their immunogenicity and efficacy.

#### 4.2.4. Conserved Sequences

Certain regions of HIV-1 are less variable than others due to a need for functional or structural conservation, and changes in these regions result in a cost to the viral fitness, or the capacity of HIV to replicate [125,126]. Therefore, targeting conserved regions may be beneficial in the design of HIV-1 vaccines, to generate both cross-reactive and potentially protective immune responses. The targeting of more conserved (Gag and Pol) compared to more variable proteins (Env) by CD8^+^ T cells in the transition from early to chronic HIV-1 infection correlates with decreased viral loads [127]. In the Step trial, Li *et al.* [128] found a bias towards the generation of more variable epitopes, with highly conserved epitopes being detected at a lower frequency than would be expected. Immunogens consisting of conserved epitopes would therefore refocus the immune response away from the usual hierarchy of responses. Furthermore, Kopycinski *et al.* [129] provided evidence that T cell responses to conserved regions of HIV-1 correlate with inhibition of HIV-1 replication. In a phase I vaccine trial testing Ad35 vectors expressing HIV-1 subtype A *gag*, *RT*, *int*, *nef* and *env*, responding participants were capable of eliciting *in vitro* suppression of viral replication of diverse HIV-1 subtypes, which correlated with targeting of conserved regions [129].

This strategy was applied by Letourneau *et al.* [130] in designing a T cell immunogen, consisting of an artificial protein containing the 14 most conserved regions of the HIV-1 proteome, derived from a consensus sequence from subtypes A, B, C and D, expressed by the three most studied HIV vaccine vectors, DNA, Adenovirus and MVA. The protein was immunogenic in mice, inducing HIV-1-specific T cell responses that produced cytokines and were capable of killing target cells [130], functional attributes associated with viral control, as discussed in 3.1. In a more recent study, Borthwick *et al.* [131] tested this approach in clinical trials. A DNA, simian adenovirus and modified vaccinia virus Ankara vaccine, delivered in a prime-boost strategy, was administered in a Phase I trial. The vaccine induced high levels of T cells that inhibited viral replication in a VIA. Viral inhibition was mediated by CD8^+^ T cells targeting Gag and Pol epitopes that are usually subdominant in natural infection [131].

It is important to note that the responses observed in HIV-infected individuals may not necessarily be the ones that should be induced by a vaccine, in the sense that immunogenicity, although related to antigenicity, can be significantly different. Studying responses in HIV-1-infected individuals can guide the identification of highly targeted epitopes that can be used in vaccine formulations, but immunodominant responses generated after vaccination may not necessarily reflect those observed in natural infections [132]. Ultimately, the potentially relevant responses that T cell-based vaccines to these immunogens should elicit can only be determined in vaccine trials.

## 5. Conclusions

It is now well accepted that an effective vaccine against HIV-1 would need to elicit both potent broadly cross-neutralizing antibodies, as well as broadly cross-reactive and cross-protective T cell responses against the vast majority of HIV-1 subtypes and recombinants, either to augment protection at the site of exposure, or potentially reduce viral load if breakthrough infection occurs. Therefore, there has been a continued focus on HIV-1 vaccine immunogen design to elicit T cell responses that can control viral replication and be cross-reactive and inhibit the full spectrum of HIV-1 diversity globally. It is likely that an approach that combines mosaic and conserved approaches will likely be superior, where the T cell response is focused on the most conserved and difficult-to-escape regions, the diversity of conserved regions is covered with mosaics, and pathways to escape are targeted through generating depth. Optimal vaccine vectors and adjuvants need to be employed to elicit such responses at sufficient potency (magnitude, functional and phenotypic quality and anatomical location) to inhibit HIV replication, not merely to cross-react with HIV. Lastly, in addition to CD8^+^ T cell responses, cross-reactive CD4^+^ responses, that are required for both the development of CD8^+^ memory and neutralizing antibody responses to HIV [133], will need to be elicited. Ultimately, such approaches need to demonstrate protection from HIV infection in human clinical trials.
